# *TUBA1A* mutation can cause a hydranencephaly-like severe form of cortical dysgenesis

**DOI:** 10.1038/srep15165

**Published:** 2015-10-23

**Authors:** Setsuri Yokoi, Naoko Ishihara, Fuyuki Miya, Makiko Tsutsumi, Itaru Yanagihara, Naoko Fujita, Hiroyuki Yamamoto, Mitsuhiro Kato, Nobuhiko Okamoto, Tatsuhiko Tsunoda, Mami Yamasaki, Yonehiro Kanemura, Kenjiro Kosaki, Seiji Kojima, Shinji Saitoh, Hiroki Kurahashi, Jun Natsume

**Affiliations:** 1 Division of Molecular Genetics, Institute for Comprehensive Medical Science, Fujita Health University, Toyoake, Japan; 2 Department of Pediatrics, Fujita Health University School of Medicine, Toyoake, Japan; 3 Department of Pediatrics, Nagoya University Graduate School of Medicine, Nagoya, Japan; 4 Laboratory for Medical Science Mathematics, RIKEN Center for Integrative Medical Sciences, Yokohama, Japan; 5 Department of Developmental Medicine, Research Institute, Osaka Medical Center for Maternal and Child Health, Izumi, Japan; 6 Department of Pediatrics, Yamagata University Faculty of Medicine, Yamagata, Japan; 7 Department of Medical Genetics, Osaka Medical Center and Research Institute for Maternal and Child Health, Osaka, Japan; 8 Department of Neurosurgery, Takatsuki General Hospital, Osaka, Japan; 9 Division of Regenerative Medicine, Institute for Clinical Research, Osaka National Hospital, National Hospital Organization, Osaka, Japan; 10 Department of Neurosurgery, Osaka National Hospital, National Hospital Organization, Osaka, Japan; 11 Center for Medical Genetics, Keio University School of Medicine, Tokyo, Japan; 12 Department of Pediatrics and Neonatology, Nagoya City University Graduate School of Medical Sciences, Nagoya, Japan

## Abstract

*TUBA1A* mutations cause a wide spectrum of lissencephaly and brain malformations. Here, we report two patients with severe cortical dysgeneses, one with an extremely thin cerebral parenchyma apparently looking like hydranencephaly and the other with lissencephaly accompanied by marked hydrocephalus, both harbouring novel *de novo* missense mutations of *TUBA1A*. To elucidate how the various *TUBA1A* mutations affect the severity of the phenotype, we examined the capacity of the mutant protein to incorporate into the endogenous microtubule network in transfected COS7 cells by measuring line density using line extraction in an immunofluorescence study. The mutants responsible for severe phenotypes were found to incorporate extensively into the network. To determine how each mutant alters the microtubule stability, we examined cold-induced microtubule depolymerisation in fibroblasts. The depolymerisation of patients’ fibroblasts occurred earlier than that of control fibroblasts, suggesting that microtubules bearing mutated tubulins are unstable. Both mutations are predicted to participate in lateral interactions of microtubules. Our data suggest that the *TUBA1A* mutations disrupting lateral interactions have pronounced dominant-negative effects on microtubule dynamics that are associated with the severe end of the lissencephaly spectrum.

In the past two decades, it has become evident that the genes encoding cytoskeletal proteins are important in the developing brain^1^. Their importance was initially inferred from the identification of genes encoding microtubule-associated proteins (MAPs), such as *LIS1* (also known as *PAFAH1B1*) and *DCX*, which are mutated in the lissencephaly spectrum^2^. Several years later, new disorders associated with mutations in genes encoding for α- or β-tubulin were described^3^,^4^,^5^,^6^,^7^,^8^,^9^,^10^. The α- and β-tubulins are the major components of microtubules and are characterised by variable isotypes whose expressions are spatially and temporally regulated^11^,^12^. Mutations in a number of neuronally expressed tubulin genes are associated with a spectrum of cortical development malformations commonly referred to as tubulinopathies. These disorders are caused by mutations in *TUBA1A*^3^, *TUBB2B*^4^, *TUBB3*^5^,^6^, *TUBB*^7^, *TUBB4A*^8^, *TUBB2A*^9^, and *TUBG1*^10^ genes. These mutations thought to involve varying degrees of abnormal neuronal proliferation, migration, and postmigrational development that result in a large spectrum of malformations, including lissencephaly, pachygyria, polymicrogyria, and microcephaly^7^,^11^,^13^.

Microtubules are ubiquitous structural components that contribute to the cytoskeleton, cilia, flagella, axon fibres, and mitotic spindles^14^. Microtubules are dynamic polymers consisting of tandem repeats of α- and β-tubulin heterodimers, which assemble in a head-to-tail fashion at the growing ends of microtubules. Each microtubule is constructed from 13 laterally connected protofilaments of repeating tubulin heterodimers; lateral interactions between each microtubule form the hollow and cylindrical microtubule body^13^,^15^.

*TUBA1A* gene encoding α1a-tubulin is expressed in almost all post-mitotic neurons throughout neuronal development. *TUBA1A*-related cortical dysgenesis typically shows a posteriorly predominant lissencephaly with cerebellar hypoplasia (LCH), dysmorphic basal ganglia, thin or absent corpus callosum, congenital microcephaly, ventricular dilatation, and abnormalities of the hippocampus and brainstem^16^. However, the clinical phenotypes caused by *TUBA1A* mutations vary considerably. Recently, *TUBA1A* mutations have also been described in perisylvian asymmetrical polymicrogyria^17^,^18^,^19^, polymicrogyria-like cortical dysplasia^20^, and microlissencephaly in foetal cases^21^. The clinical manifestations of affected patients often include congenital microcephaly, severe intellectual disability, neurodevelopmental delay with diplegia or tetraplegia, and epilepsy^22^.

In our study, we performed whole-exome sequencing of two patients with severe cortical dysgeneses. One patient had an extremely thin cerebral parenchyma apparently looking like hydranencephaly, whereas the other had lissencephaly accompanied by marked hydrocephalus. We identified two novel *de novo* heterozygous *TUBA1A* mutations, c.190 C>T (p.R64W) and c.74 G>T (p.C25F). In addition, we performed a functional assay of the mutant proteins to determine why these patients show more severe phenotypes than patients with classical lissencephaly.

## Results

### Patients’ characteristics

Patient 1 (NCU_F41) was a 3-year-old girl. She was born at a gestational age of 37 weeks by caesarean section. Her parents were healthy and unrelated. Her elder sister was also healthy and had normal development. Her mother was referred to our hospital for foetal growth restriction, microcephaly, and marked ventricular dilatation of her foetus on ultrasonography from 28 weeks of gestation. At patient delivery, the amniotic fluid was excessive but the placenta and umbilical cord were normal. Her Apgar scores were 3 and 5 at 1 and 5 min, respectively. She could not breathe spontaneously and needed mechanical ventilation. Her birth weight was 2116 g (–2.0SD), head circumference was 29.6 cm (–2.4SD), and body length was 44 cm (–1.8SD). She had microcephaly, microphthalmos, widely spaced eyes, and micrognathia. Truncal hypotonia with spastic tetraplegia was evident and her digital joints were contractured.

**Figure 1:**
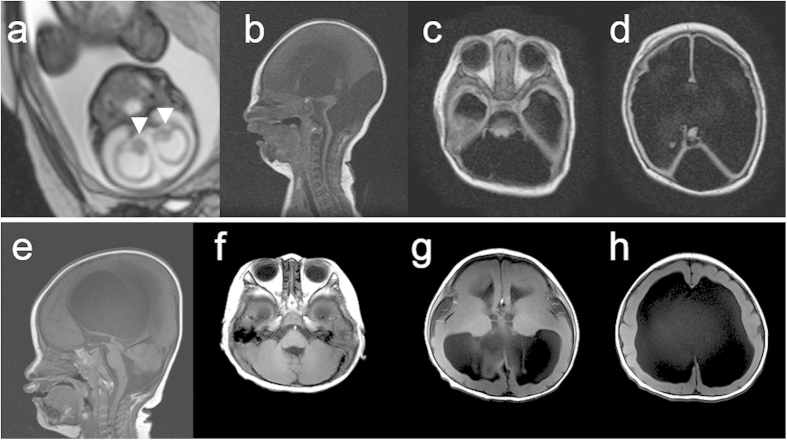
Brain MRI findings of two patients with mutations in *TUBA1A*. (**a**) Foetal MRI at 28 weeks of gestation of patient 1 (T2-weighted image). White arrowheads indicate the basal ganglia. (**b**–**d**) MRI findings at 6 days after the birth of patient 1 (**b**: T1-weighted image; **c**,**d**: Fluid Attenuated Inversion recovery [FLAIR]). The patient showed an extremely thin cerebral parenchyma, hypoplastic brain stem, and agenesis of the cerebellum and corpus callosum. Most of the intracranial space was occupied with cerebrospinal fluid. (**e–h**) MRI findings at 1 year of age of patient 2 (T1-weighted images). The patient showed marked ventricular dilatation with a thin cortex, agyria, or limited pachygyria, poorly differentiated dysmorphic basal ganglia, agenesis of the corpus callosum, and slightly hypoplastic cerebellar vermis. The brain stem appeared to be normal.

An ophthalmologic examination revealed bilateral optic nerve hypoplasia. Foetal MRI at 28 weeks of gestation and brain MRI at 6 days after birth revealed an extremely thin cerebral parenchyma, hypoplastic brain stem, and agenesis of the cerebellum and corpus callosum (Fig. 1a–d). Test results for toxoplasma, rubella, cytomegalovirus, and herpes simplex (TORCH) infections were negative. Her karyotype was normal 46, XX. After birth, she presented with focal clonic seizures, sometimes with oxygen desaturation. Her electroencephalogram showed extremely poor background activities and focal rhythmic delta waves during the seizures. As the seizures were treated with phenobarbital, they were partially controlled.

At the age of 1 month, a tracheotomy and tracheal separation were performed because of her recurrent aspiration pneumonia. A gastrostomy and fundoplication were also done at the same time. Because of the agenesis of the pituitary, trichlormethiazide for central diabetes insipidus and hydrocortisone and levothyroxine for hypopituitarism were needed. Since her head circumference had been gradually enlarged, a ventriculoperitoneal shunt was placed at the age of 7 months to control the head growth and to assist in nursing care. At 3 years of age, she had spastic tetraplegia and no definite awareness of her environment.

We performed whole-exome sequencing of peripheral blood DNA from the patient and both her parents (Supplementary Fig. S1a). A *de novo* heterozygous c.190 C>T (p.R64W) variant was identified in *TUBA1A*. Subsequent Sanger sequencing confirmed the presence of this variant in the patient 1 and absence in the genomes of both her parents (Supplementary Fig. S2). The c.190 C>T variant was predicted to be damaging by both PolyPhen-2 and SIFT. No potentially pathogenic variants related to malformations of cortical development were identified in any other genes in patient 1 (Supplementary Table S1).

Patient 2 (K3373) was a 2-year-old boy. Ventricular dilatation was identified in the foetal period. His parents were healthy and unrelated. His elder sister was also healthy and had normal development. He was born at a gestational age of 39 weeks by vaginal delivery. His Apgar scores were 8 and 9 at 1 and 5 min, respectively. His birth weight was 2792 g (−0.9SD), head circumference was 33 cm (–0.2SD), and body length was 49.5 cm (+0.3SD). After birth, he was diagnosed with lissencephaly (Fig. 1e–h). He could breathe and swallow by himself. At the age of 8 months, he began suffering from epileptic spasms, which could be controlled with sodium valproate and zonisamide. At 2 years of age, he had spastic tetraplegia and was unable to roll over. Although he would not make eye contact and did not speak, he could react to sound and had a smile when his mother called his name.

We performed whole-exome sequencing of peripheral blood DNA from the patient and both his parents (Supplementary Fig. S1b). A *de novo* heterozygous c.74 G>T (p.C25F) variant was identified in *TUBA1A* and confirmed by Sanger methods (Supplementary Fig. S2). This variant was not detected in the genomes of both his parents by Sanger sequencing. The c.74 G>T variant was predicted to be damaging by both PolyPhen-2 and SIFT. There were no potentially pathogenic variants related to malformations of cortical development in any other genes in patient 2 (Supplementary Table S1). Both *TUBA1A* mutations of patients 1 and 2 are located at the amino acids which are conserved across many species (Supplementary Fig. S1c).

### Structural modelling of TUBA1A mutations

**Figure 2:**
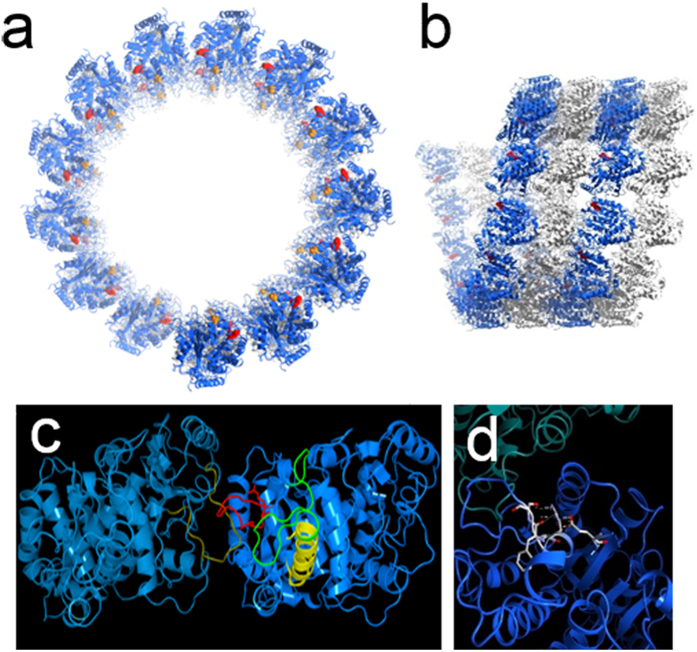
Three-dimensional mapping of *TUBA1A*-mutated residues in a microtubule structure. (**a**) End-on view of a microtubule structural model. A microtubule consists of 13 longitudinal protofilaments that are connected via lateral interactions. α-tubulin molecules are blue, β-tubulin molecules are white, R64 residues are red, and C25 residues are orange. (**b**) Side view of a microtubule. C25 residues are not shown in this view because they are located on the luminal side of a microtubule. (**c**) Higher resolution image of the lateral interaction between the α-tubulin molecules. Both light blue and dark blue are α-tubulin molecules. The M loop is gold, the H1′-S2 loop is red, the H1-H1′ loop is green, and the helix H1 is yellow. The R64 and C25 residues have side chains in this figure. (**d**) The R64 residue forms hydrogen bonds with surrounding residues, E3, F53, and S54.

Both *TUBA1A* mutations reported here are located in the N-terminal domain (Supplementary Fig. S1d) and are predicted to be associated with lateral interactions between microtubules (Fig. 2a–c). According to structural studies conducted using cryoelectron microscopy, protofilaments in microtubules are primarily connected between the M loops and the H1′-S2 and H2-S3 loops^15^. R64 is located on the H1′-S2 loop of α-tubulin (Fig. 2c), which participates in lateral interactions. R64 forms hydrogen bonds with the surrounding residues, E3, F53, and S54 (Fig. 2d). These hydrogen bonds may be involved in the structural stability and/or flexibility of the lateral interactions. Therefore, the R64W mutation is predicted to directly disrupt the lateral interaction. C25 is located on the boundary between helix H1 and the H1-H1′ loop of α-tubulin (Fig. 2c), and this residue faces the luminal side of microtubules (Fig. 2a). The H1-H1′ loop appears to support the H1′-S2 loop and the H2-S3 loop to enhance lateral interactions^15^. Therefore, the C25F mutation may secondarily compromise lateral interactions.

### TUBA1A mutants alter the ability of α-tubulin to incorporate into the microtubule network

**Figure 3:**
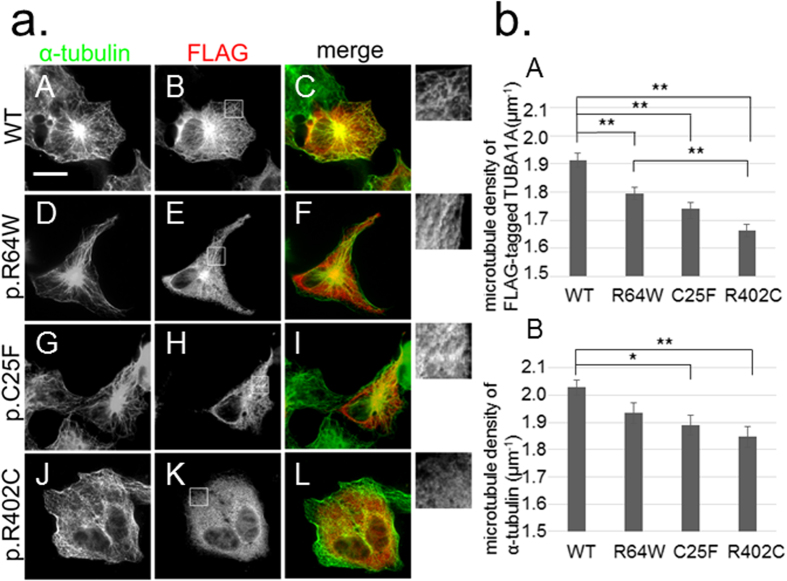
The ability to incorporate into the microtubule network varies among TUBA1A wild-type and mutants. (**a**) Transfected COS7 cells were examined by immunofluorescence using an anti-FLAG antibody (red) and an anti–α-tubulin antibody (green). C-terminal FLAG-tagged wild-type TUBA1A was visualised as lines (B) and colocalised with the cytoskeleton of α-tubulin (C). In the case of FLAG-tagged mutant TUBA1A, there were fewer lines than with the wild-type (E,H,K). Insets are magnified images of the boxes. Scale bar, 20 μm. (**b**) Quantification of microtubule density. We extracted linear staining of FLAG-tagged TUBA1A (A) and the overall cytoskeleton network of α-tubulin (B) of each cell by using the ImageJ KBI Line Extract plug-in and calculated the line density of each cell. Bars represent the means ± SEM (32 cells from wild-type, 28 cells from R64W, 28 cells from C25F, and 31 cells from R402C). Asterisks indicate statistically significant differences (one-way ANOVA and Tukey’s post-hoc test; *p < 0.05, **p < 0.01). (A) The microtubule density of FLAG-tagged mutant TUBA1A was significantly lower than that of FLAG-tagged wild-type TUBA1A. The microtubule density of R64W was the highest among the mutants and that of R402C was the lowest. (B) The microtubule density level of α-tubulin followed the same order as that of FLAG-tagged TUBA1A.

We examined the ability of the TUBA1A mutants to incorporate into the endogenous microtubule network (Fig. 3). We generated constructs designed to express TUBA1A mutants upon transfection of COS7 cells. The constructs were for wild-type, p.R64W, and p.C25F, as well as p.R402C, which is a recurrent *TUBA1A* mutation that expresses the phenotype of classical lissencephaly, similar to *LIS1* mutations^23^. Transfected cells were examined by immunofluorescence using an anti-FLAG antibody to detect the expression of the transgene and an anti–α-tubulin antibody to detect the overall microtubule network. At 24 h post-transfection, we found that FLAG-tagged wild-type TUBA1A was visualised as lines (Fig. 3a,B) and colocalised with the α-tubulin cytoskeleton (Fig. 3a,C), suggesting that FLAG-tagged wild-type TUBA1A could incorporate into the microtubule network. On the other hand, we found that FLAG-tagged mutant TUBA1A was visible not only as lines, but also as puncta that were diffusely distributed throughout the cytoplasm (Fig. 3a,E,H,K). The linear staining merged with α-tubulin, but the puncta did not colocalise with α-tubulin (Fig. 3a,F,I,L), suggesting that some of the mutant TUBA1A protein could not incorporate into the microtubule network. We observed more incorporated FLAG-tagged TUBA1A protein with R64W and C25F transfection than with R402C. To exclude the influence of the acidic charges of the FLAG tag, we also examined the incorporation of the Myc-tagged TUBA1A protein. Consistent with the data of the FLAG-tagged protein, the incorporation into the microtubule network of Myc-tagged mutant TUBA1A was less than wild-type (Supplementary Fig. S4).

To quantify the incorporation of mutant protein in each transfected cell, we determined the linear staining of FLAG-tagged TUBA1A of each cell by using the ImageJ KBI Line Extract plug-in for line extraction^24^ (Supplementary Fig. S3a). These lines indicated the microtubule network of incorporated FLAG-tagged TUBA1A. After line extraction, we measured the total length of the lines and calculated the line density of each cell. The microtubule density of FLAG-tagged TUBA1A for each mutant transfection was analysed.

We found that the microtubule density of FLAG-tagged mutant TUBA1A was significantly lower than that of FLAG-tagged wild-type TUBA1A (Fig. 3b,A). Among TUBA1A mutants, the microtubule density of R64W was the highest, whereas that of R402C was the lowest. We also calculated the microtubule density of α-tubulin in the same manner (Fig. 3b,B). We found that the microtubule density level followed the same order as that of FLAG-tagged TUBA1A. Therefore, the amount of the overall microtubule network tended to depend on the amount of incorporated overexpressed TUBA1A protein. These data indicated that R64W tended to permit higher levels of microtubule incorporation than C25F and R402C. Because there were no significant differences in the mean relative FLAG intensities, the expression levels of FLAG-tagged protein in the analysed cells were thought to be similar among wild-type and mutants (Supplementary Fig. S3b).

To assess microtubule dynamics in transfected COS7 cells, we examined repolymerisation after cold induced depolymerisation (Supplementary Fig. S5). There were significantly less cells containing the asters of α-tubulin in the cases of R64W transfection than wild-type (Supplementary Fig. S5c).

### TUBA1A mutants alter microtubule stability

**Figure 4:**
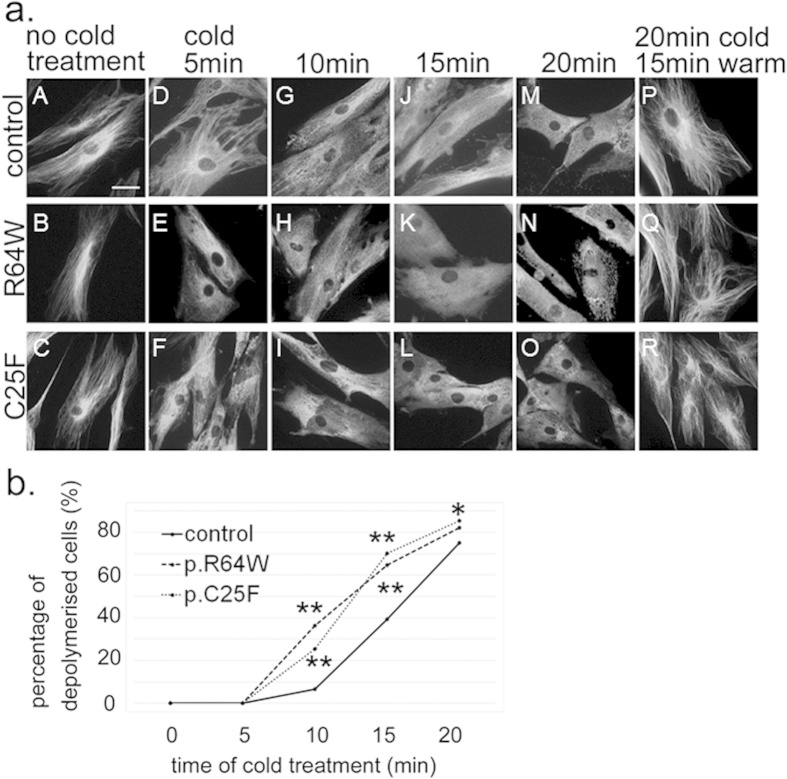
Microtubule behaviour and stability in patients’ fibroblasts after various periods of cold treatment. (**a**) Before cold treatment, the cytoskeleton morphologies were similar among control and mutant fibroblasts (A–C). Note that fibroblasts of R64W and C25F were depolymerised after 15 min of cold treatment, whereas polymerised microtubules were still present in control fibroblasts (J–L). Scale bar, 20 μm. (**b**) Percentage of completely depolymerised cells after cold treatment of 0, 5, 10, 15, and 20 min. We counted completely depolymerised cells among 275–400 cells in each condition. After 10 min of cold treatment, the percentage of R64W and C25F fibroblasts showing depolymerisation was more than four times that of control fibroblasts. Asterisks indicate statistically significant differences compared with control (Fisher’s exact test and Bonferroni correction using numbers of cells with and without complete depolymerisation, *p < 0.05, **p < 0.0001).

We investigated microtubule behaviour in patients’ fibroblasts to assess the effects of these mutations on the microtubule stability (Fig. 4). Microtubules of fibroblasts start to depolymerise when they are incubated on ice. We examined the cytoskeleton morphology and the depolymerised tubulin in the fibroblasts by immunofluorescence staining of α-tubulin (Fig. 4a). We first showed by RT-PCR that *TUBA1A* was actually expressed in fibroblasts of the patients and control fibroblasts (control_1) (Supplementary Fig. S6). We defined a cell that contained no linear staining of α-tubulin at 400× magnification to be a completely depolymerised cell. We compared the percentage of completely depolymerised cells between patients’ fibroblasts and control ones when they were incubated on ice for 0, 5, 10, 15, and 20 min (Fig. 4b). There were no completely depolymerised control or mutant cells after 0 and 5 min of cold treatment (Fig. 4a,A–F). After 10 min of cold treatment, we found that the percentage of completely depolymerised cells from the R64W and C25F patients was more than four times that of the control (Fig. 4a,G–I,b). The differences for comparison R64W and C25F cells with control cells were statistically significant (*p *= 5.6 × 10^−24^ and *p* = 5.8 × 10^−14^, respectively, Fig. 4b). This suggested that the depolymerisation of patients’ fibroblasts occurred sooner than the control ones. There were almost no differences in the percentage of depolymerised cells after 20 min of cold treatment (Fig. 4a,M–O,b). Repolymerisation of the microtubules could occur in control and mutant fibroblasts after treatment on ice for 20 min and subsequently at 37 °C for 15 min (Fig. 4a,P–R) . These data suggested that mutated microtubules are less stable than normal ones.

To assess an underlying mechanism by which *TUBA1A* mutations lead to cortical dysgeneses, we examined the mitosis and the migration of the patients’ fibroblasts (Supplementary Fig. S7, S8). There were no significant differences between the patients’ and control fibroblasts both in the mitosis and in the migration.

## Discussion

In our present study, we identified two novel heterozygous missense *TUBA1A* mutations in patients with severe cortical dysgeneses, one with an extremely thin cerebral parenchyma apparently looking like hydranencephaly and the other with lissencephaly accompanied by marked hydrocephalus. *TUBA1A*-related cortical dysgenesis typically shows a posteriorly predominant lissencephaly with cerebellar hypoplasia (LCH), dysmorphic basal ganglia, thin or absent corpus callosum, congenital microcephaly, ventricular dilatation, and abnormalities of the hippocampus and brainstem^16^. Without these characteristics, it is difficult to determine whether a case with certain brain malformations is a *TUBA1A*-related disorder using only MRI findings. The brain MRI of our patient harbouring the *TUBA1A* p.R64W mutation manifested an extremely thin cerebral parenchyma with severe hydrocephalus, agenesis of the cerebellum and the corpus callosum, and hypoplastic brain stem, the most severe form of brain malformations. It looked like hydranencephaly but was distinguished from hydranencephaly by the existence of an extremely thin cerebral parenchyma. The cerebral cortex was too thin to assess the layer structure using brain MRI. Thus, our data indicate that the spectrum of *TUBA1A*-related brain malformations is broader than expected.

All previously reported, *TUBA1A* mutations have been heterozygous missense mutations^25^,^26^. The presence of missense mutations and the absence of nonsense mutations, frameshifts, or whole gene deletions suggest that the mutation results in gain-of-function or has a dominant-negative effect, rather than haploinsufficiency. Among tubulinopathies, it has been proposed that the severity of nervous system impairments may depend on the relative abundance of mutant α- and β-tubulin heterodimers compared with wild-type, combined with their ability to incorporate into the microtubule cytoskeleton, which affect dynamics, motor protein, or MAP interaction in different dominant-negative fashions^13^.

In the case of *TUBB3* mutations, R262H substitution permits much more heterodimer formation and microtubule incorporation than R262C, both *in vitro* and in mammalian cells. R262C results in isolated eye movement restrictions, whereas R262H causes not only severe eye movement restrictions, but also other neurological impairments and brain malformations^6^. In the same way, the recurrent R402H mutation in *TUBA1A*, which causes a more severe lissencephaly with complete agyria than R402C, produces a mutant protein that permits higher levels of heterodimer formation and microtubule incorporation than R402C^23^,^27^. We showed that R64W had the highest amounts of mutant protein incorporation into the network compared with the other mutants and caused the most severe phenotype. In the repolymerisation experiments, the expression of TUBA1A R64W protein impaired the ability of the endogenous α-tubulin to repolymerise. Thus, a greater extent of mutant protein incorporation into the endogenous microtubule network may result in more severe phenotypes in a dominant-negative fashion.

In the current study, the data of the incorporation of FLAG-tagged R402C TUBA1A was different from the previous reports^23^,^27^. The reason of the difference could be based on the position of the FLAG tag. To exclude the influence of the acidic charges of the FLAG tag, we also examined the incorporation of the Myc-tagged TUBA1A protein. Consistent with the data of the FLAG-tagged protein, limited incorporation of the R402C mutation was observed and the data did not depend on the type of tags. According to the previous report^27^, the R402C mutation generated tubulin heterodimers in significantly reduced yield *in vitro* folding reaction. It may be consistent that the R402C mutation disrupts αβ heterodimerisation, leading to little incorporation into the microtubule network.

Tubulin contains three separate structural domains, N-terminal, intermediate, and C-terminal^28^(Supplementary Fig. S1d). These three domains participate in five distinct functions: heterodimer stability, longitudinal and lateral protofilament interactions, nucleotide exchange and hydrolysis, and microtubule–protein interactions^13^. In neurons, lateral interactions are particularly important because microtubules are arranged in dense networks and must be resistant to forces that cause bending or buckling to maintain their structural integrity^13^,^15^,^29^. Mutations found at positions essential for lateral interactions are predicted to impede the polymerisation and dynamic properties of microtubules, resulting in microtubules that may be relatively nondynamic or unstable and more likely to depolymerise^13^. In cold-induced depolymerisation of fibroblasts, it is consistent that the two TUBA1A mutants, R64W and C25F, could make microtubules less stable than those of the controls by disrupting lateral interactions.

We investigated the severity of *TUBA1A*-related cortical dysgeneses that are associated with lateral interactions in previous reports as well as in this report. *TUBA1A* mutations that primarily participate in lateral interactions include L286F^30^ (M loop), E55K^31^, T56M^25^, R64W (H1′-S2 loop), and L92V^23^ (H2-S3 loop). The patients harbouring L286F, T56M, and L92V were foetuses. Using the classification of Kumar *et al*.^23^, four mutations (L286F, T56M, R64W, and L92V) are classified in the severe lissencephaly with cerebellar hypoplasia (LCH severe group 4). On the other hand, E55K belongs to the moderate lissencephaly (LIS moderate group 1) with extreme microcephaly. There are also neighbouring loops that may support lateral interactions. The H1-H1′ loop supports the H1′-S2 loop and the H2-S3 loop, and the end of H6 and the S9-S10 loop appear to stabilise the M loops^15^. *TUBA1A* mutations that secondarily participate in lateral interactions include C25F, E27Q^26^ (H1-H1′ loop), Y210C^17^, D218Y^23^ (the end of H6), G366R^32^, A369T^25^, and V371E^25^ (S9–S10 loop). Five mutations (E27Q, Y210C, D218Y, G366R, and V371E) were classified into the LCH severe group 4 and C25F resembled the LCH severe group 4 phenotype except for milder cerebellar vermis hypoplasia. Only A369T showed central pachygyria. Therefore, many of the reported *TUBA1A* mutations at positions associated with lateral interactions caused severe phenotypes of brain malformations (LCH severe group 4).

In the case of β-tubulin, R62 of TUBB3 is also positioned in the H1′-S2 loop of β-tubulin and is predicted to participate in lateral interactions^6^. However, patients harbouring R62Q have a milder phenotype among TUBB3 mutations. As it was reported that residues involved in lateral interactions cluster in regions of divergence between species and marked difference between α- and β-tubulins^28^, there may be different consequences for α- and β-tubulins when lateral interactions are disrupted. We propose that the differences in clinical manifestations among tubulinopathies caused by a mutated tubulin gene should be evaluated.

In conclusion, our data suggest that mutations in *TUBA1A* at positions essential for lateral interactions may lead to severe phenotypes of brain malformations. However, we did not show the consequences of these mutants for neuronal developmental processes such as proliferation, migration, differentiation, and axonal guidance. In patients’ fibroblasts, the mitosis and the migration were not impaired. Further studies of how each mutant affects microtubule function and how it impairs neuronal developmental processes will reveal the precise function of microtubules in normal neuronal development.

## Methods

### Patients

Genetic testing was approved by the ethical committees of Fujita Health University and collaborated institutes in accordance with the principles of the Declaration of Helsinki, and the Ethical Guidelines for Human Genome/Gene Analysis Research by the Ministry of Education, Culture, Science, and Technology, the Ministry of Health, Labor, and Welfare, and the Ministry of Economy, Trade, and Industry of Japan. Blood samples from affected individuals and their parents, and skin biopsy samples were obtained with informed consent according to local institutional review board guidelines.

### Whole-exome sequencing and validation

Genomic DNA was extracted from peripheral blood using the QIAamp DNA Blood Midi Kit according to the manufacturer’s instructions (Qiagen, Tokyo, Japan). Three micrograms of DNA were sheared into 150–200-bp fragments using theM220 Focused-ultrasonicator (Covaris, Woburn, MAUSA). To capture the exonic DNA, we used the SureSelect XT Human All Exon V5 capture library (Agilent Technologies, Santa Clara, CAUSA). We then constructed a sequence library using the SureSelect XT Target Enrichment System for the Illumina Paired-End Sequencing Library kit (Agilent Technologies) and performed DNA sequencing of 100-bp paired-end reads using the Illumina HiSeq 2000 sequencer. The sequencing data were mapped to the reference genome (GRCh37/hg19) using BWA (ver.0.6.1). Variant calling was performed using SAMtools (ver.0.1.16) and GATK (ver.1.6) software as previously reported^33^. To identify disease causative mutations, we excluded known variants found in public databases (dbSNP138, 1000 Genomes Project, NHLBI ESP6500, and Exome Aggregation Consortium [ExAC]) and a control in-house database, except for those also identified as pathogenic mutations in the NCBI ClinVar and HGMD databases. We focused on non-synonymous single nucleotide variants (SNVs), insertions and deletions (indels), and splice site variants. Predictions of possible impact of amino acid substitution on the structure and function by variant were performed using PolyPhen-2^34^ and SIFT^35^ software. The mutations were confirmed by Sanger sequencing.

### Structural modelling of TUBA1A mutations

Tubulin dimer structure (PDB ID:1JFF) docked into the density map (MT-13-3, EMDB ID: EMD-5193) using Chimera (https://www.cgl.ucsf.edu/chimera/) after the homology modelling of missing residues 35–60 in α-tubulin was kindly provided by Dr. Haixin Sui, New York State Department of Health^15^. Polymerised microtubule images were then generated with Chimera and MolFeat (FiatLux, Tokyo, Japan).

### Transfection experiments

The full-length cDNA encoding the human *TUBA1A* sequence was generated by PCR using a template from placenta cDNA (Forward primer: 5′-TAAGCGGCCGCCATGCGTGAGTGCATCTCCATCCAC-3′, Reverse primer: 5′-TAACTGCAGGTATTCCTCTCCTTCTTCCTCACCCTC-3′). The PCR product was cloned into the *Not* I and *Pst* I sites of the pCMV-4A vector (Agilent Technologies), which is a mammalian expression vector for tagging proteins with a C-terminal FLAG (DYKDDDDK) epitope under the control of the CMV promoter. Three mutations—p.R64W, p.C25F, and p.R402C—were generated by PCR using a template of the wild-type construct. For construction of the Myc tag vectors, the *Xho* I and *Apa* I fragment of these FLAG tag constructs were replaced with a synthesized DNA encoding a Myc tag (The fragment sequence is 5′-TCGAGGAACAAAAACTCATCTCAGAAGAGGATCTGTAGGGCC-3′). All constructs were checked by DNA sequencing.

Constructs were transfected into COS7 cells grown on glass coverslips in Opti-MEM I using Lipofectamine 2000 (Life Technologies). At 24 h after transfection, cells were fixed with ice-cold methanol and stained with a mouse monoclonal anti–α-tubulin antibody (Santa Cruz Biotechnology) and a goat polyclonal anti-DYKDDDDK antibody (Novus Biologicals) at dilutions of 1:250 and 1:5000, respectively. Secondary antibodies were donkey anti-mouse IgG Alexa Fluor 488 and donkey anti-goat IgG Alexa Fluor 594 (Life Technologies) at dilutions of 1:1000. In repolymerisation experiments of COS7 cells^35^, cells were incubated on ice for 30 min and then restored to 37 °C for 1.5 min. Cells were immediately fixed with ice-cold methanol and stained as above. For the Myc tag experiment, a rabbit polyclonal anti-Myc antibody (MBL) at dilution of 1:100 and donkey anti-rabbit IgG Alexa Fluor 594 (Life Technologies) at dilutions of 1:1000 were used.

### RT-PCR

RT-PCR was done by manufacturer’s protocol. NucleoSpin RNA II (MACHEREY-NAGEL) and SuperScript III First-Strand Synthesis System (Life Technologies) were used. The primer sequences of *TUBA1A* and *HPRT* are shown in the Supplementary Figure S6.

### Depolymerisation experiments of fibroblasts

Fibroblasts were derived from skin biopsies taken from the patients with p.R64W and p.C25F mutations and control people. The depolymerisation experiments were done as previously described^5^. Briefly, fibroblasts (passage 3) grown on glass coverslips in IMDM containing 10% foetal bovine serum, penicillin 50 U/ml and streptomycin 50 ug/ml were incubated for 0, 5, 10, 15, and 20 min on ice and fixed. Repolymerisation experiments of fibroblasts were performed by incubating cells on ice for 20 min and then restoring them to 37 °C for 15 min. Cells were stained with a mouse monoclonal anti–α-tubulin antibody used at a dilution of 1:100. The secondary antibody was donkey anti-mouse IgG Alexa Fluor 488 used at a dilution of 1:1000.

### Mitotic Index and mitotic spindle formation

Fibroblasts (passage 5) grown on glass coverslips were fixed with ice-cold methanol and stained with a mouse monoclonal anti–α-tubulin antibody and a rabbit polyclonal anti-phospho Histone H3 (pH3) (Ser10) antibody (MILLIPORE) at dilutions of 1:250 and 1:100, respectively. Secondary antibodies were donkey anti-mouse IgG Alexa Fluor 488 and donkey anti-rabbit IgG Alexa Fluor 594 (Life Technologies) at dilutions of 1:1000. The pH3 signal-positive mitotic fibroblasts were counted from at least 830 total cells.

### *In vitro* scratch assay

The *in vitro* scratch assay was done as previously described^37^. For each image, distances between one side of scratch and the other were measured using ImageJ and 50 readings of distances were measured for each sample.

### Image acquisition

Slides were observed under a fluorescence microscope (Axio Imager M1; Carl Zeiss) equipped with a digital camera (AxioCam HRc; Carl Zeiss). COS7 cells and fibroblasts were observed at 630× and 400× magnifications, respectively. *In vitro* scratch assay, dishes were observed under Axiovert 200M (Carl Zeiss) equipped with a digital camera (AxioCam MRm; Carl Zeiss) and fibroblasts were observed at a 100× magnification. Images were captured with the same settings using AxioVision 4.8 software (Carl Zeiss).

### Quantification of microtubule density and relative FLAG intensity

Quantification of microtubule density was performed as previously described^24^. For each COS7 cell, a region of interest (ROI) was manually assigned along the edge of the cell by the free hand tool in ImageJ. We then calculated the area and the mean intensity of the ROI by using the ImageJ ROI manager. We used the KBI Line Extract plug-in for line extraction. The parameters were giwsIter 5, mdnmsLen 20, pickup above 0.0, shaven Len 5, and del Len 5. The total length of the extracted lines was calculated by the KBI Line Feature plug-in in ImageJ for the α-tubulin staining and the FLAG staining. The total length of the extracted lines in a selected cell divided by the area of the cell gave the microtubule density. These plug-ins are available as part of the KBI ImageJ plug-in package (http://hasezawa.ib.k.u-tokyo.ac.jp/zp/Kbi/ImageJKbiPlugins). The relative FLAG intensity was determined in ImageJ by subtracting the mean red signal intensity of an untransfected cell from that of a transfected cell. The untransfected cell was in the same image as that of the transfected cell.

### Statistical analysis

One-way ANOVA and Tukey’s post-hoc analysis and a chi-square test were performed using IBM SPSS Statistics Version 22. Fisher’s exact test and the Bonferroni correction were performed using R software. We considered *p* < 0.05 to be significant after correction.

## Additional Information

**How to cite this article**: Yokoi, S. *et al*. *TUBA1A* mutation can cause a hydranencephaly-like severe form of cortical dysgenesis. *Sci. Rep*. **5**, 15165; doi: 10.1038/srep15165 (2015).

## Supplementary Material

Supplementary Information

Supplementary Table S1
