# Supplementary material for: TUBA1A mutation can cause a hydranencephaly-like severe form of cortical dysgenesis
Source: Sci Rep. 2015 Oct 23;5:15165. doi: 10.1038/srep15165 (PMC4615979; doi:10.1038/srep15165)
Supplement: Supplementary Information [file srep15165-s1.pdf]

TUBA1A mutation can cause a hydranencephaly-like severe form of cortical dysgenesis

Setsuri Yokoi, Naoko Ishihara, Fuyuki Miya, Makiko Tsutsumi, Itaru Yanagihara, Naoko Fujita, Hiroyuki Yamamoto, Mitsuhiro Kato, Nobuhiko Okamoto, Tatsuhiko Tsunoda, Mami Yamasaki, Yonehiro Kanemura, Kenjiro Kosaki, Seiji Kojima, Shinji Saitoh, Hiroki Kurahashi & Jun Natsume

Supplementary Figure S1

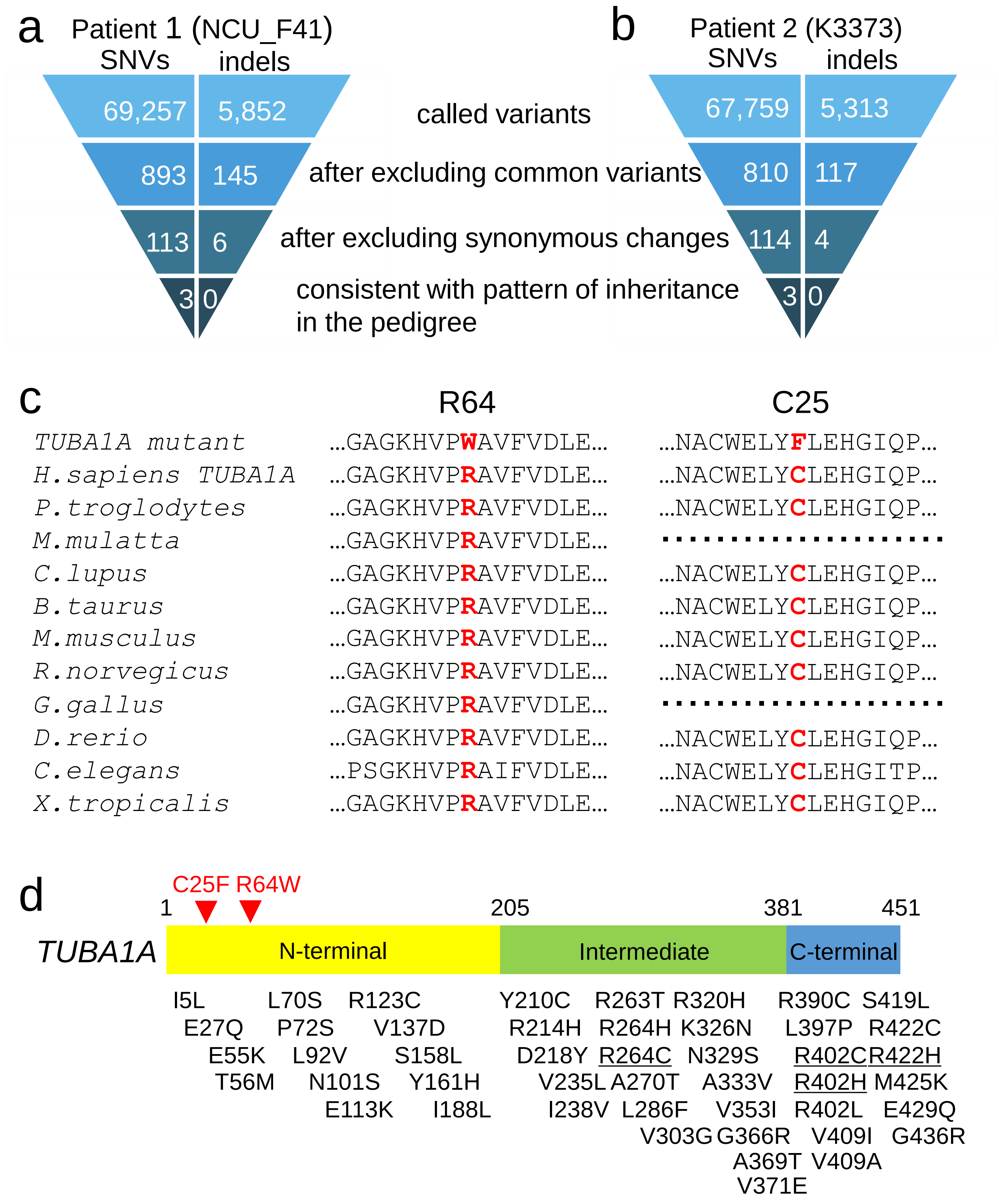

Supplementary Figure S1

(a) The filtering steps for determining candidate mutations in NCU_F41 (patient 1). The top row is the number of called variants by next-generation sequencing. The second row is the number of variants after filtering out known variants in databases, except for those that were also known pathogenic mutations. The third row is the number of variants after excluding synonymous change variants. The bottom row is the number of variants consistent with the phenotype in the pedigree (i.e., the total number of the autosomal recessive, X-linked recessive, *de novo*, and compound heterozygous variants). (b) The filtering steps for determining candidate mutations for the K3373 (patient 2). (c) Phylogenetic alignments for *TUBA1A* variations in this study. Mutant tubulin amino acid sequences are aligned against corresponding wild-type and equivalent tubulin homologues in other phylogenetic species. (d) Schematic representation of the functional domains of *TUBA1A* and mutations associated with previously reported malformations of cortical development[25,26]. Recurrent mutations of *TUBA1A* are underlined.

Supplementary Figure S2

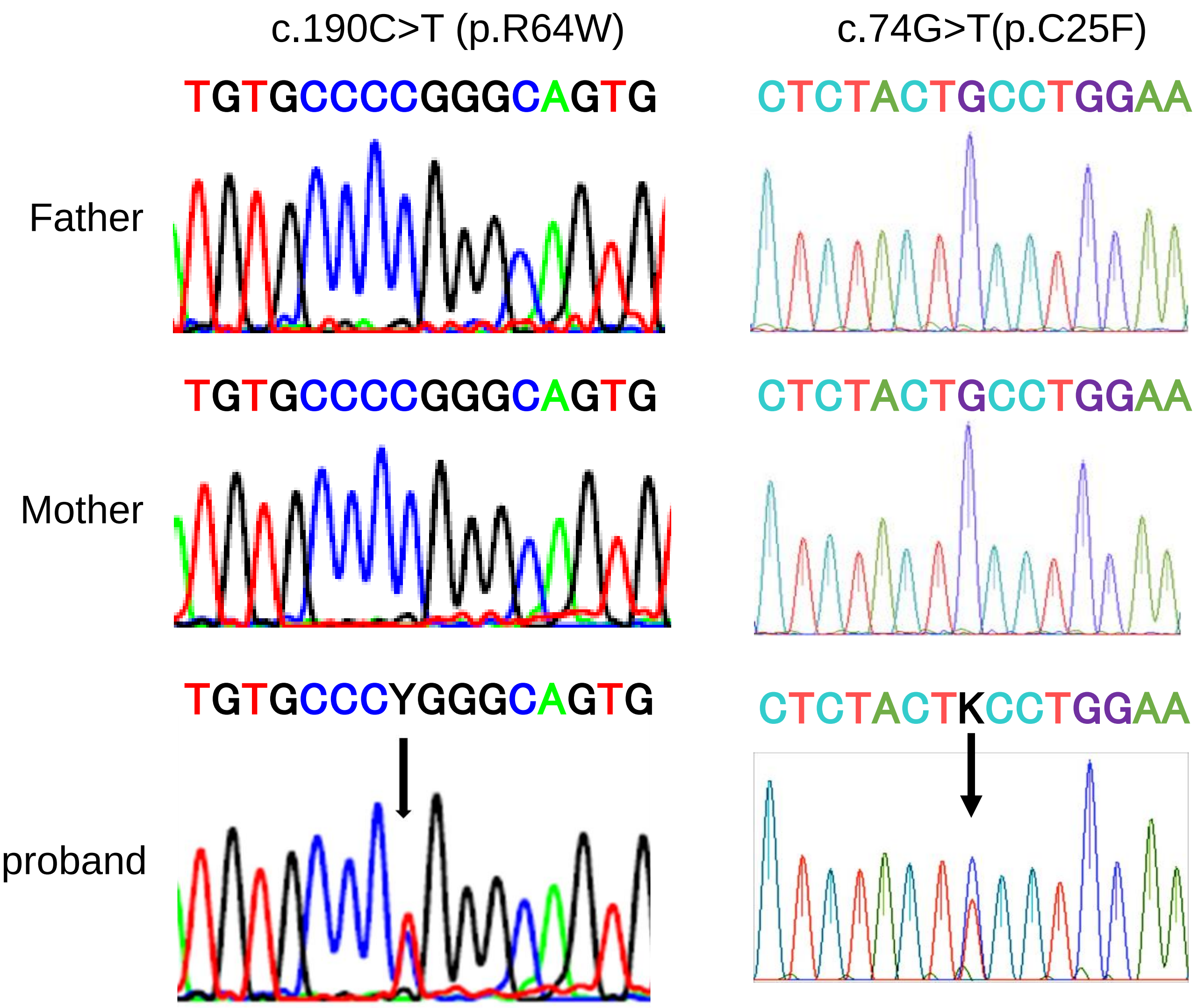

Supplementary Figure S2

Validation of the *TUBA1A* mutations by Sanger sequencing. The missense mutations, c.190C>T (p.R64W) and c.74G>T (p.C25F), are shown. These variants are absent in the genomes of both their parents.

Supplementary Figure S3

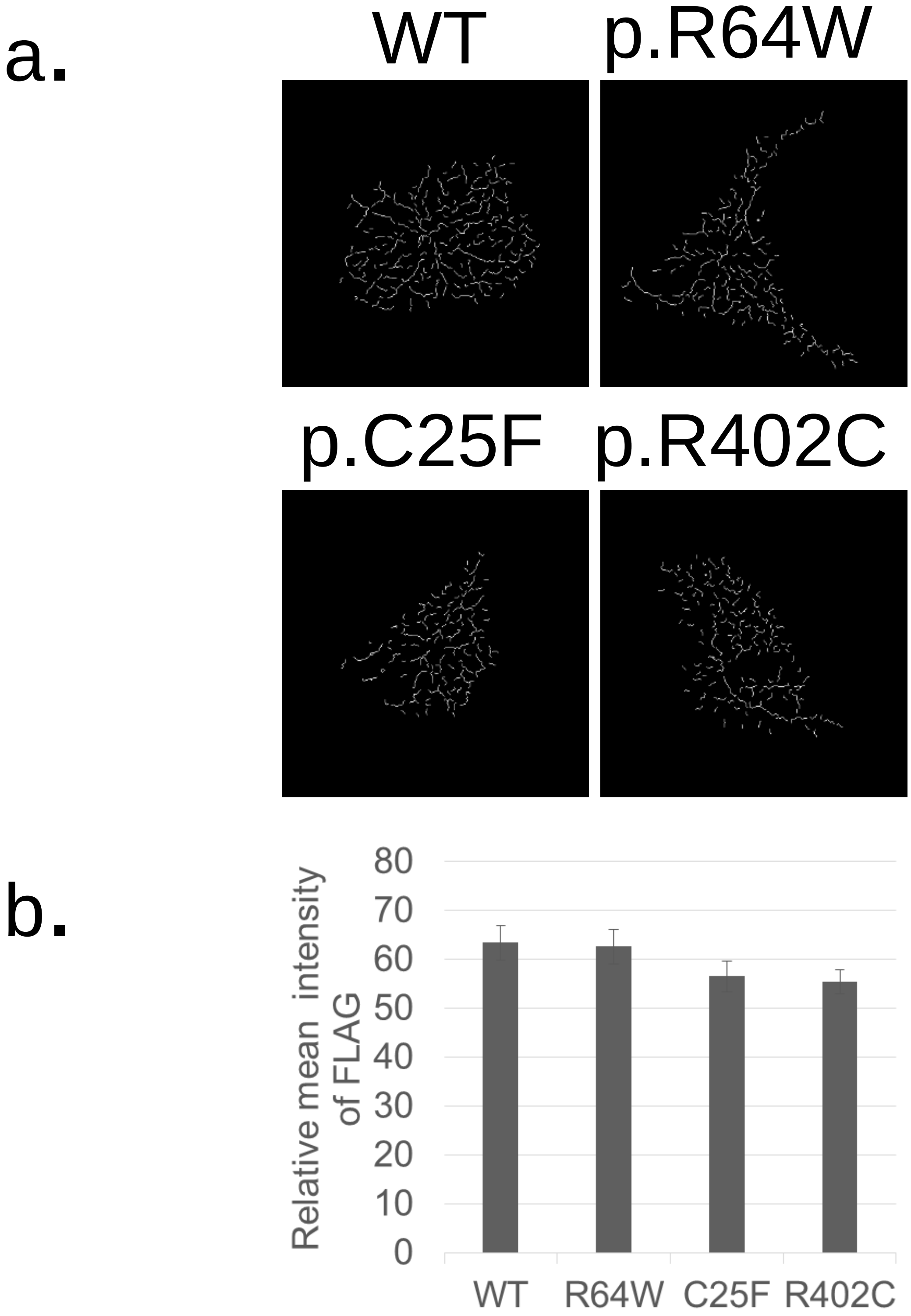

Supplementary Figure S3

(a) The extracted lines of the cells in Fig.3 obtained using the ImageJ KBI Line Extract plug-in. (b) Relative mean FLAG intensity of the analysed cells. Bars are the means ± SEM (32 cells from wild-type, 28 cells from R64W, 28 cells from C25F, and 31 cells from R402C). There were no statistically significant differences among WT and mutants.

Supplementary Figure S4

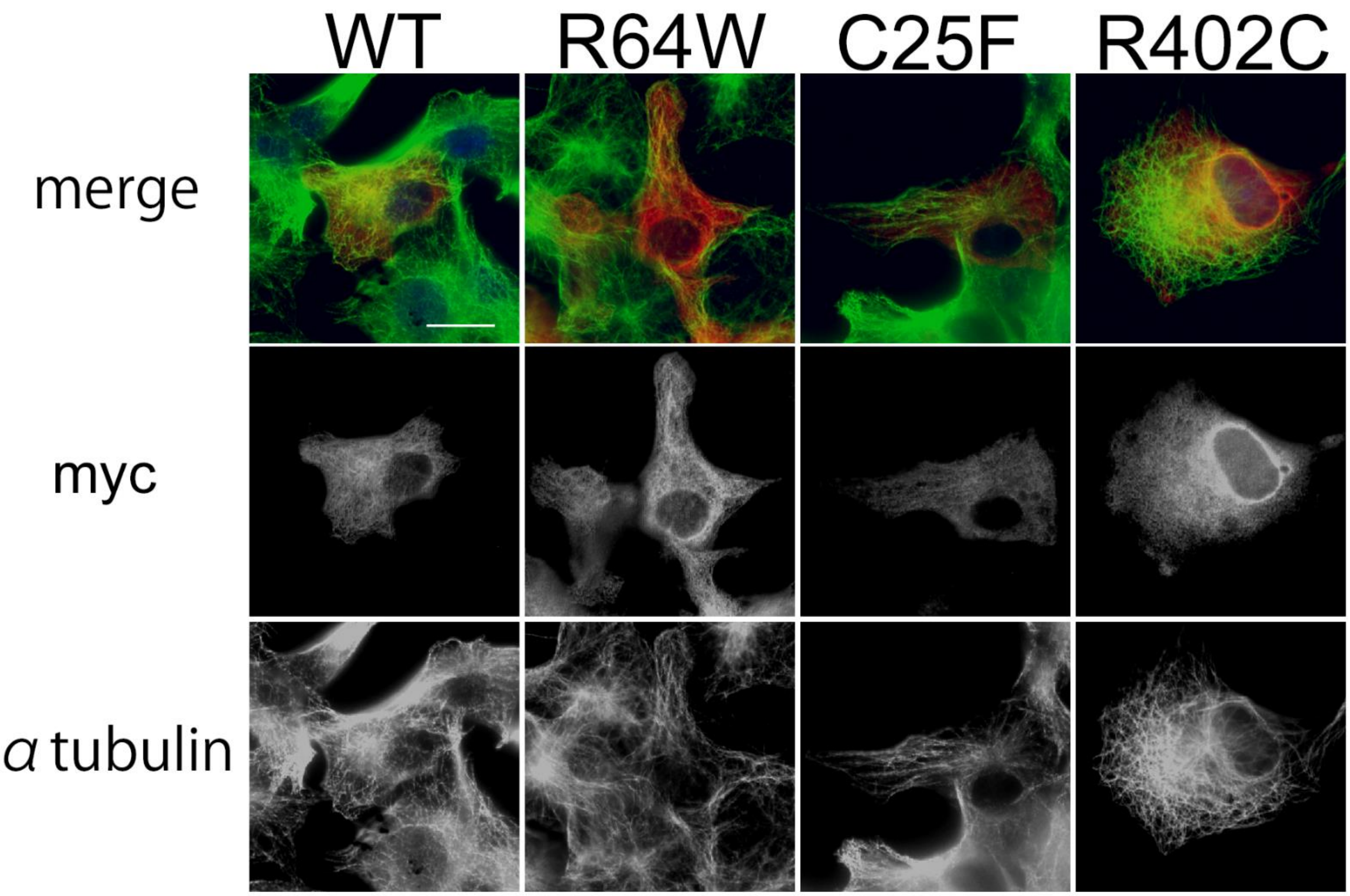

Supplementary Figure S4

Representative images of myc-tagged TUBA1A. Myc-tagged wild-type TUBA1A was visualized as lines, suggesting that myc-tagged wild-type TUBA1A could incorporate into the endogenous microtubule network. Myc-tagged mutant TUBA1A could incorporate into the network to some extent. We observed more incorporated myc-tagged TUBA1A protein with R64W and C25F transfection than with R402C. Scale bar, 20μm.

Supplementary Figure S5

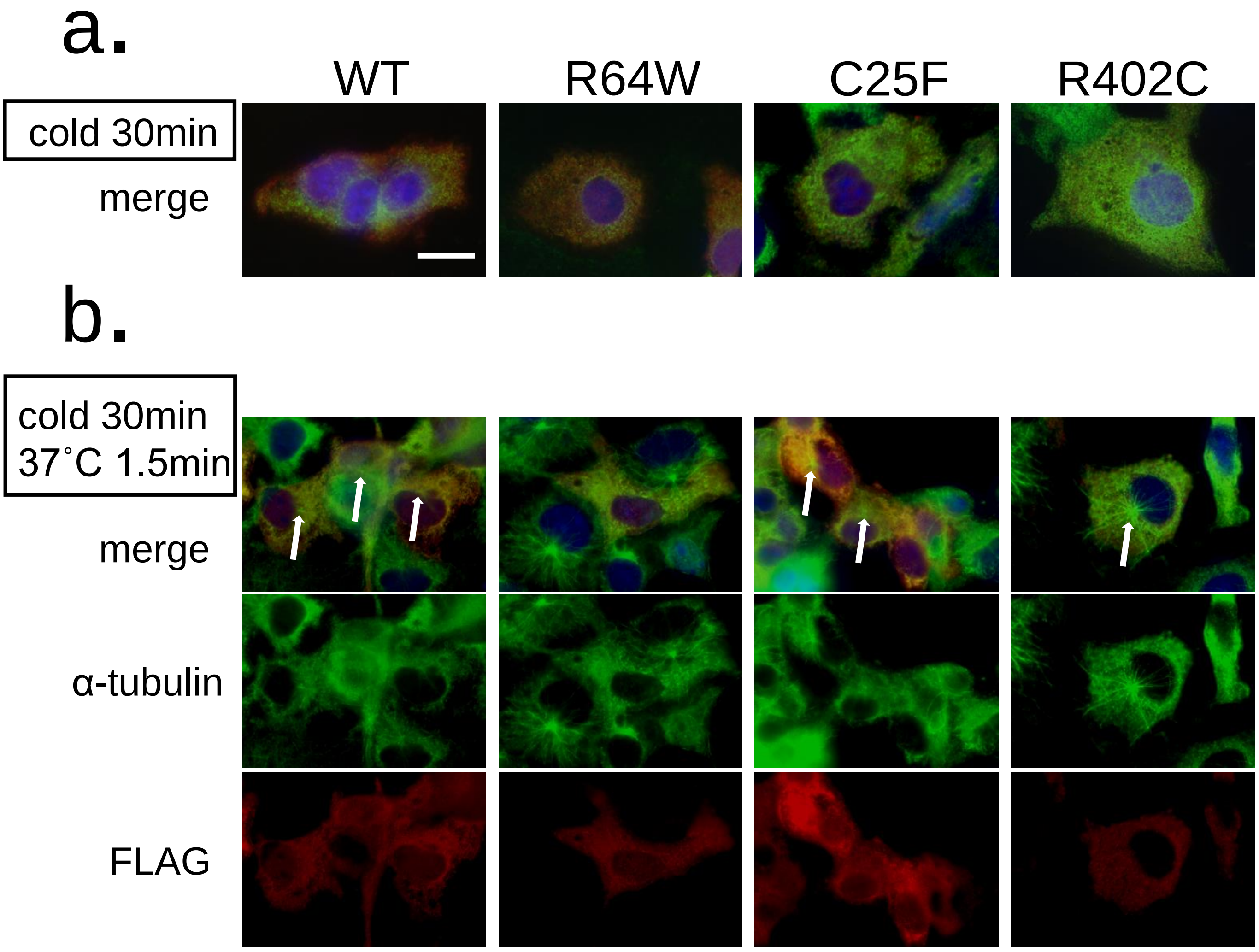

c.

	WT	R64W	C25F	R402C
cells containing the asters of α-tubulin (total 100 cells)	86	62	76	83

* (WT vs R64W)

Supplementary Figure S5

Repolymerisation after cold-induced depolymerisation. Scale bar, 20μm. (a) Transfected COS7 cells were incubated on ice for 30 min. Microtubules were completely depolymerised. (b) Cells were restored to 37˚C 1.5 min after 30min cold treatement. Microtubules started to repolymerise from the asters (white arrows). (c) The number of cells containing the asters of α-tubulin. In the case of p.R64W, only 62% of the cells contained the asters of α-tubulin after 1.5min at 37˚C, compared to 86% in wild-type-transfected cells. The difference for comparison R64W cells with control cells was statistically significant (*p*=0.00052, Fisher's exact test and Bonferroni correction).

Supplementary Figure S6

a.

TUBA1A_forward : CACGTCGCTTGCACCAATCAC
TUBA1A_reverse : GAGGACACAATTTGACCTATTAACC
HPRT_forward : CTTCCTCCTCCTGAGCAGTC
HPRT_reverse : AACACTTCGTGGGGTCCTTT

b. TUBA1A_forward

```
TUBA8  GGGC----------------------CCGGCG--------CGCCTGTCCC---------- 71
TUBA3D AGGA---------------------GGTTGCA------GTTGGGCGCTCA---------- 132
TUBA3E AGGT---------------------CATTGCA------GTTGGGCGCTCA---------- 40
TUBA3C GGGA---------------------GGCTGTC------GTTGGGCGTGC----------- 42
TUBA1A TACCTCATCCCACGTC---------GCTTGCACCAATCACCAGTC-TCCT---------- 136
TUBA1C GAGT--GCTTTGTGT----------GCTTGGA------ATTAG--ATCCT---------- 100
TUBA1B CAGCGACCGCGGCACC---------GCCTGTGC------CCGCCCGCCCC---------- 83
TUBA4A TA---------------------------GCG--------CAG---TTCT---------- 36
TUBAL3 ------------------------------------------------------------
```

TUBA1A_reverse

```
TUBA8  ACCTATACCAACCTCAACCGCCTCATCAGTCAGATTGTGTCCTCAATCACTGCTTCTCTC 905
TUBA3D ACGTACACCAACCTCAATCGCCTGATTGGGCAGATCGTGTCCTCCATCACAGCCTCCCTG 919
TUBA3E ACGTACACCAACCTCAATCGCCTGATTGGGCAGATCGTGTCCTCCATCACGGCCTCCCTG 827
TUBA3C ACGTACACCAACCTCAATCGCCTGATTGGGCAGATCGTGTCCTCCATCACGGCCTCCCTG 831
TUBA1A ACCTATACTAACCTGAATAGGTTAATAGGTCAAATTGTGTCCTCCATCACTGCTTCCCTG 1071
TUBA1C ACCTACACTAACCTTAACCGCCTTATTAGCCAGATTGTGTCCTCCATCACTGCTTCCCTG 964
TUBA1B ACCTACACTAACCTTAACCGCCTTATTAGCCAGATTGTGTCCTCCATCACTGCTTCCCTG 947
TUBA4A ACCTACACCAACCTCAATCGCCTCATTAGCCAAATTGTCTCCTCCATCACAGCTTCTCTG 814
TUBAL3 TCTCATGCCAGCATCAATAGATTGGTGGTTCAGGTGGTATCTTCCATCACTGCCTCCCTC 785
        *  *  * * * * **  *  *  *    **  * ** ** ** ***** ** ** **
```

c.

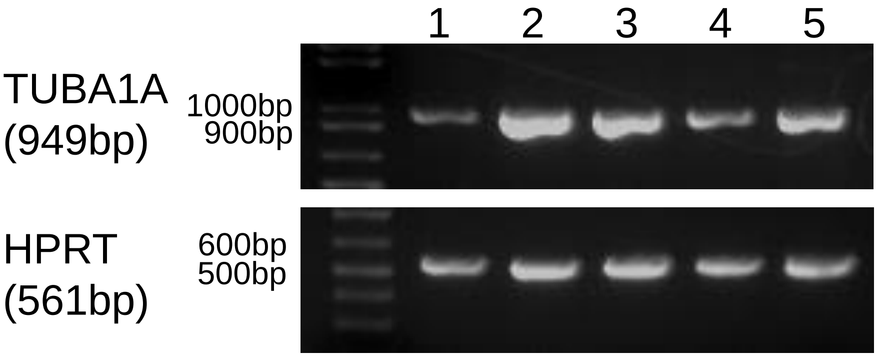

Supplementary Figure S6

(a) Primer sequences are shown. (b) A part of α-tubulin isoform cDNA aligned by ClustalW. Primer sequences were shown in red. Accession no. are TUBA8 NM_018943.2, TUBA3D NM_080386.3, TUBA3E NM_207312.2, TUBA3C NM_006001.2, TUBA1A NM_006009.3, TUBA1C NM_001303114.1, TUBA1B NM_006082.2, TUBA4A NM_006000.2, TUBAL3 NM_024803.2. (c) RT-PCR using the primers specific for *TUBA1A*. Lane 1, control_1 fibroblast; lane 2, N811; lane 3, N836; lane 4, C25F; lane 5,R64W.

Supplementary Figure S7

a.

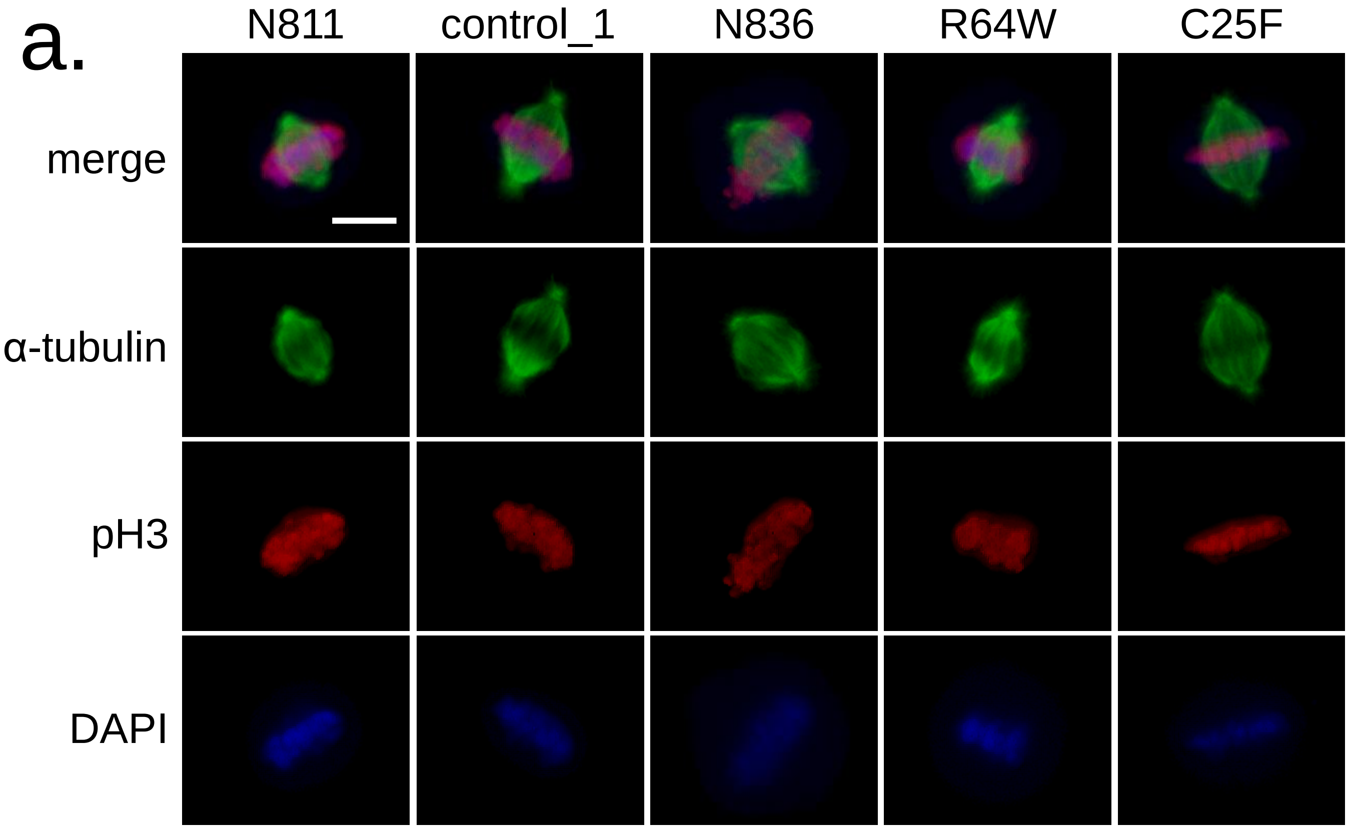

b.

	R64W	C25F	control_1	N836	N811
mitotic cell/total cell	9/858	9/874	10/867	18/851	11/832
mitotic index	1.05	1.03	1.15	2.12	1.32

Supplementary Figure S7

(a) Representative images of mitotic spindle formation in fibroblasts. pH3, phosphorylated histone H3 (marker of mitotic chromatin). Scale bar, 10μm. (b) Mitotic index of fibroblasts. Mitotic cells were identified as pH3 positive cells by immunofluorescense. A chi-square test was used to compare mitotic index. There are no statistically significant differences between each group (p=0.256).

Supplementary Figure S8

a.

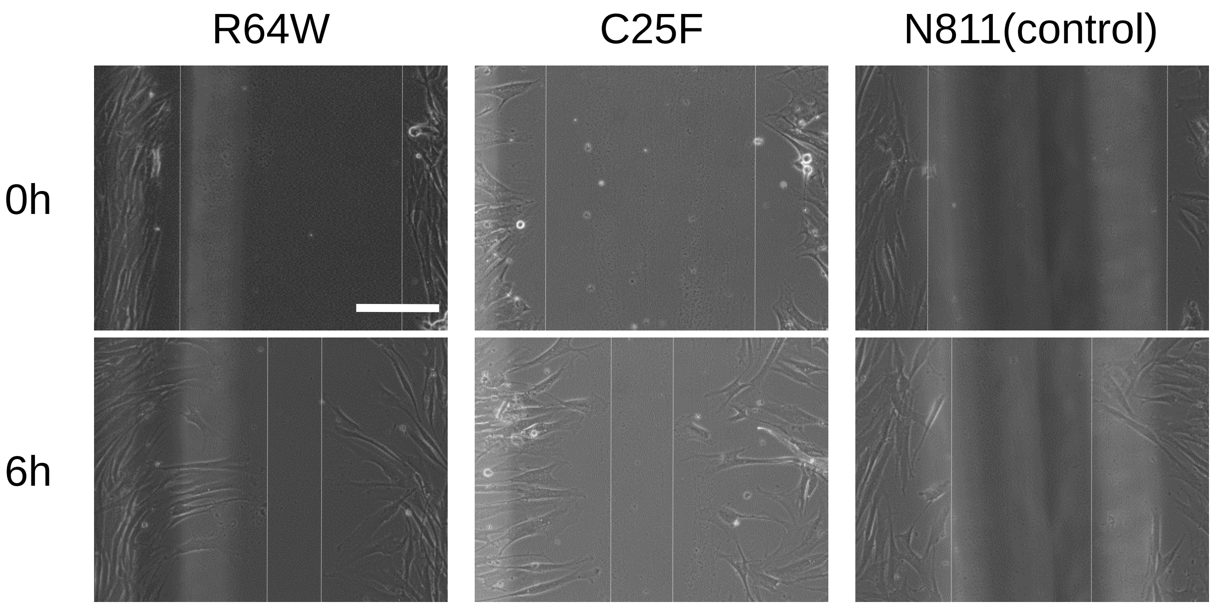

b.

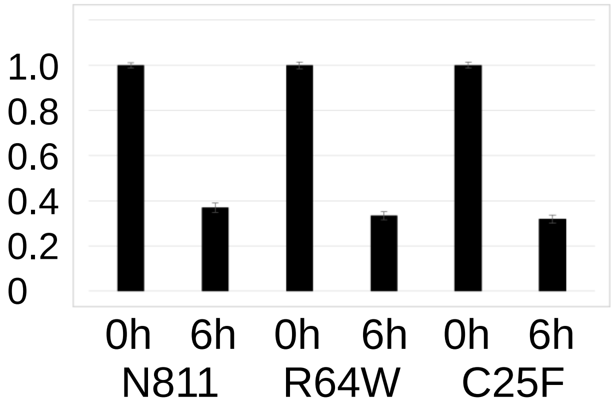

Supplementary figure S8

(a) Analysis of passage 5 fibroblasts migration by *in vitro* scratch assay. Images were acquired at 0 and 6 h after scratch. Dotted lines defined the margins of fibroblasts. Distances between one side of scratch and the other were measured. Scale bar; 200μm. (b) The ratio of the average distances of 6h to that of 0h. The differences for comparison R64W and C25F cells with N811 cells were not statistically significant (p = 0.346 and p = 0.144, respectively; one-way ANOVA and Tukey's post-hoc test).
